# Metabolic rate and insulin-independent glucose uptake increase in a TDP-43^Q331K^ mouse model of amyotrophic lateral sclerosis

**DOI:** 10.1016/j.heliyon.2025.e42482

**Published:** 2025-02-05

**Authors:** Tanya S. McDonald, Cedric S. Cui, Titaya Lerskiatiphanich, Jianina Marallag, John D. Lee

**Affiliations:** School of Biomedical Sciences, The University of Queensland, St Lucia, Brisbane, QLD, 4072, Australia

**Keywords:** Amyotrophic lateral sclerosis, Glucagon, Glucose tolerance, Insulin, Metabolic rate

## Abstract

Impaired glucose regulation is increasingly recognised in amyotrophic lateral sclerosis (ALS), yet the precise mechanisms remain unclear. Here, we investigated energy balance and glucose control in TAR DNA-binding protein 43 (TDP-43)^Q331K^ mice, a model of ALS, at both the early and late symptomatic stages of disease. Mutant TDP-43^Q331K^ mice and non-transgenic controls underwent indirect calorimetry, as well as intraperitoneal glucose, insulin, and glucagon tolerance testing. We also examined plasma hormone levels and quantified α- and β-cell areas in pancreatic islets. Throughout disease progression, TDP-43^Q331K^ mice exhibited elevated metabolic rates, with a transient increase in food intake at the early stages. At the later stages of disease, heightened glucose uptake was observed despite unchanged insulin secretion or tolerance, indicating mechanisms independent of insulin. Notably, TDP-43^Q331K^ mice maintained fasting blood glucose levels even when circulating glucagon levels were reduced, suggesting that alternative pathways contribute to preserving euglycemia. These findings reveal a distinct metabolic profile in TDP-43^Q331K^ mice, underscoring the complexity of glucose dyshomeostasis in ALS.

## 1 Introduction

Amyotrophic lateral sclerosis (ALS) is a neurodegenerative condition characterised by muscle weakness, impaired motor function, and atrophy and denervation of skeletal muscle, resulting from the degeneration of motor neurons in the spinal cord and motor cortex [1]. The etiology of ALS is complex, with several mechanisms reported to be involved in the onset and progression of disease, including neuroinflammation, protein aggregation and dysfunction in energy metabolism [2,3].

Common features in both sporadic and familial cases of ALS include an inability to maintain weight, increased resting energy expenditure and rapid weight loss, which are all associated with poorer disease outcomes in patients [[4], [5], [6]]. A higher body mass index at the onset of disease is also associated with an increase in survival [7]. Furthermore, treatments of high calorie and high fat diets have been shown to be somewhat beneficial in patients and mouse models of ALS [8,9], indicating that metabolic disturbances can alter disease progression in ALS. Insulin resistance is also reported to play a role in disease progression in both animal models and patients with ALS. Specifically, there are reports that diabetes mellitus increases the risk of ALS in people [10,11]. Although evidence of insulin resistance has been reported in ALS [12,13], there are also reports that conflict these finding. For example, prior findings suggest that type 2 diabetes may delay the onset of ALS, or that insulin tolerance is unchanged [[14], [15], [16]]. Thus, further investigation is required to understand how energy homeostasis and insulin signalling is affected in ALS.

We previously showed that symptomatic SOD1^G93A^ mice display increased energy expenditure and enhanced uptake of exogenous glucose through insulin-independent pathways [17]. Furthermore, these mice were shown to be glucagon intolerant. As SOD1 transgenic mice represent one model of familial ALS, in this study, we aimed to extend these findings by profiling the metabolic perturbations in the TDP-43^Q331K^ transgenic mouse model of ALS, using a whole-body approach. Among the various ALS models, we selected the TDP-43^Q331K^ mouse model as it harbors a mutation associated with familial ALS and closely mimics the neuropathological and metabolic hallmarks observed in human ALS cases. This model exhibits progressive neurodegeneration linked to TDP-43 pathology, a common feature in both familial and sporadic ALS, providing a relevant platform for studying alterations in metabolism and glucose homeostasis. Additionally, the CNS-specific expression of mutant TDP-43 allows us to isolate the contributions of CNS degeneration to ALS-associated metabolic dysregulation, reducing potential confounding effects from peripheral expression. This model, therefore, offers a valuable tool for investigating how neurodegenerative processes impact systemic metabolism and glucose regulation in ALS.

Similar to the SOD1^G93A^ mice, TDP-43^Q331K^ mice exhibited increased metabolic rate and glucose uptake during the later stages of disease. Interestingly, this increase in glucose uptake appeared to be driven by insulin-independent mechanisms as we found no changes in insulin concentrations or insulin sensitivity. Additionally, despite losing circulating glucagon, TDP-43^Q331K^ mice were able to maintain normal fasting plasma glucose levels, suggesting an alternate mechanism regulating blood glucose levels in these ALS mice.

## 2 Materials and methods

### 2.1 Ethical statement

All animal procedures were approved by the University of Queensland Animal Ethics Committee (199/17), following institutional and governmental regulations in Australia. The study's design and reporting also adhered to the ARRIVE guidelines.

### 2.2 Animals

Transgenic mouse strains that express TDP-43^WT^ (Line 96) or TDP-43^Q331K^ (Line 103) on the C57BL/6J background were obtained from the Jackson Laboratory (Bar Harbor, Maine, USA). These transgenic lines, along with non-transgenic (WT) controls, were generated following the procedures described previously [18]. Both TDP-43^WT^ and TDP-43^Q331K^ mice harbor a myc-tagged human TDP-43 cDNA, which is driven by the mouse prion protein promoter. It is modified in the TDP-43^Q331K^ group to contain a Q331K mutation, which has been linked to familial ALS. This protein promotor ensures that the expression of the transgene is primarily confined to the CNS, with little expression in peripheral tissues. Notably, the prion protein promoter has been shown to drive widespread expression of the transgene throughout the CNS, including regions such as the cortex, hippocampus, cerebellum, spinal cord, and hypothalamus [19]. Thus, we infer that the mutant TDP-43^Q331K^ is expressed in the hypothalamus, which is relevant for interpreting the observed metabolic effects, as the hypothalamus plays a crucial role in regulating glucose homeostasis. Male mice were used in this study to avoid any variability in energy metabolism due to hormonal fluxes throughout the oestrous cycle. Two pre-determined age groups were examined across the experiments: 40 week old (defined as early symptomatic), where TDP-43^Q331K^ mice have previously been shown to present with the initial signs of motor deficit (decline in grip-strength); and 80 week old (defined as late symptomatic), where mice display a significant weakness in hindlimb strength [18]. No significant loss in grip strength was observed in TDP-43^WT^ mice at either 40 or 80 weeks, in contrast to the decline seen in TDP-43^Q331K^ mice. This finding aligns with the expected phenotype of TDP-43^WT^ mice, which do not exhibit motor deficits associated with ALS [18]. The mouse colony was housed at the University of Queensland Biological Resources Animal Facilities. Mice were caged on Purachip bedding under a 12-h light/dark cycle (lights on at 06:00 and off at 18:00) with *ad libitum* access to food and water unless noted otherwise. Cohort sizes were determined via power calculations, drawing on data from previous SOD1^G93A^ ALS mouse studies [17]. Our primary outcome was to assess substrate tolerance over time based on the area under the curve (AUC). Based on our previous studies the standard deviation of the AUC is approximately 250 mmmol/L × 120 min. With 80 % power at a 5 % level of significance, we would be able to detect a difference of 300 mmmol/L × 120 min with 4 animals in each group.

### 2.2 Indirect calorimetry

WT and TDP-43^Q331K^ mice were individually housed and acclimated to metabolic chambers (TSE Systems, Bad Homburg, Germany) for one week before data collection. They were then monitored for 48 h in a Phenomaster open-circuit indirect calorimetry system, with data from the final 24 h used for analysis [20]. Food consumption, locomotor activity (X, Y axes), and respiratory gases (O₂, CO₂) were recorded every 30 min, and energy expenditure plus the respiratory exchange ratio (RER) were calculated using CaloSys software (TSE Systems). Throughout the study, mice were housed on a 12-h light/dark cycle (lights on at 06:00, off at 18:00) with *ad libitum* food and water. Body composition was measured using a Bruker Minispec LF50 Analyzer (7.5 MHz, Bruker Optics Inc, Billerica, MA, USA) following published methods [17]. Afterwards, mice received intraperitoneal anesthesia (zolazepam, 50 mg/kg; xylazine, 12 mg/kg), and tissues were harvested, frozen on dry ice, and stored at −80 °C. Average daily energy expenditure was adjusted for total and lean mass using ANCOVA, according to NIDDK MMPC guidelines (www.mmpc.org/shared/regression.aspx).

### 2.4 Glucose/insulin tolerance tests and glycogen challenge

Mice were handled daily for a week prior to tolerance tests to reduce stress. Intraperitoneal glucose tolerance tests (ipGTT) were performed following previously reported methods [17]. After an overnight fast (16 h), baseline blood glucose levels were measured using an Accu-Chek Guide glucometer (Roche Diabetes Care, NSW, Australia). Each mouse then received an intraperitoneal injection of 200 mg/mL D-glucose (2 mg/kg; 10 μL/g). Blood glucose levels were measured at four time points post-injection (15, 30, 60, and 120 min) by collecting 50 μL of blood from the tail vein using lithium-heparin–coated capillary tubes (Cat# 40B501, Thermo Fisher Scientific, VIC, Australia). The blood samples were centrifuged at 2000×*g* for 10 min at 4 °C, and the plasma was stored at −80 °C for subsequent hormone analysis.

Mice were allowed to recover one week before running the intraperitoneal insulin tolerance (ipITT) test. During this time, the mice were handled daily to acclimate them to the procedure. Before the test, mice were fasted for 6 h and then injected intraperitoneally with a 0.05 IU/mL human insulin solution (Cat# I9278, Sigma-Aldrich, St Louis, MO, USA), prepared in saline at a dose 0.5 IU/kg (10 μL/g body weight). Blood collection and glucose measurements were performed at the same timepoints as for the ipGTT.

Following the ipITT, mice recovered for one week before undergoing a glucagon challenge. Mice were fasted for 4 h during the early light cycle. Blood glucose concentrations were measured prior to the intraperitoneal injection of 25 μg/mL synthetic glucagon (Cat# G2044, Sigma-Aldrich, St Louis, MO, USA), prepared in saline at a dose of 250 μg/kg. Blood glucose concentrations were then measured at 10-, 20-, 30- and 60-min post-injection. Food and water were returned to the cages at the end of each experiment.

### 2.5 Plasma hormone analysis

Plasma insulin and glucagon levels were measured by a mouse insulin ELISA Kit (RRID: AB_2783626, Crystal Chem, IL, USA) and a mouse glucagon ELISA kit (RRID: AB_2811007, Crystal Chem, IL, USA), respectively. The experimental procedures were performed as per manufacturer's manual. Plates were read using a Tecan Spark plate reader (Tecan, Mannedorf, CHE).

### 2.6 Plasma epinephrine and norepinephrine analysis

Plasma epinephrine and norepinephrine levels were measured using a modified protocol [21]. Briefly, 0.5 μL of plasma was carefully applied onto a clean glass slide and allowed to dry completely under vacuum dryer. A freshly prepared 4.4 mM solution of 4-(anthracene-9-yl)-2-fluoro-1-methylpyridin-1-ium iodide (FMP-10, Cat# T1001, Tag-on, Uppsala, Sweden), in 70 % acetonitrile was then uniformly sprayed over the dried plasma samples. This application was performed using a Sunchrom SunCollect Sprayer under the following conditions: nitrogen pressure of 2.5 psi, a line distance of 2 mm, and a sprayer z-position of 35 mm. The spraying speed was set to 10 μL/min for the first layer, 20 μL/min for the second, and 40 μL/min for subsequent layers, for a total of 35 passes.

Mass spectrometry imaging (MSI) analysis was conducted using a trapped ion mobility spectrometry-time-of-flight (TIMS-TOF) Flex-MALDI-2 instrument (Bruker Daltonics, Germany), with laser power optimisation performed at the beginning of each session, typically between 70 and 75 %. Instrument calibration was achieved using an external electrospray ionisation low (ESI-L) tune mix (Agilent G1969-85000) in ESI mode. The TIMS TOF Flex was operated using TIMS Control 4.1.13 and Flex Imaging 7.2 software. The acquisition parameters were configured as follows: a single beam laser was used with a beam scan set to 46 μm in both the X and Y dimensions, resulting in a pixel size of 50 μm. Each pixel received 300 laser pulses at a frequency of 10,000 Hz. The mass-to-charge ratio (*m*/*z*) range was set from 160 to 3000. The ion source-induced dissociation (isCID) energy was set to 0 eV, ion energy to 5 eV, and collision energy to 10 eV, with the focus feature enabled.

Post-acquisition, MSI data was recalibrated using the FMP-10 matrix ion as a lock mass within the Data Analysis 6.2 software. The refined data was then imported into SCILS LAB MVS 2024a for further analysis. Dataset normalisation was achieved using root mean squared normalisation. The ion intensities of specific *m*/*z* values, corresponding to the derivatised epinephrine and norepinephrine in each plasma droplet, were subsequently exported.

### 2.7 Liver glycogen and triglyceride concentrations

Glycogen and triglyceride levels in the liver were measured using glycogen and triglyceride assay kits (Cayman Chemical, MI, USA). Tissue samples were prepared according to the manufacturer's instructions, and endpoint readings were taken using a FlexStation 3 plate reader (Molecular Devices, CA, USA). The protein content was normalised using a BCA protein assay kit (Thermo Fisher, MA, USA).

### 2.8 Pancreas immunofluorescence

Pancreatic tissue from 80-week-old male WT and TDP-43^Q331K^ mice was fixed for 2 h in 4 % paraformaldehyde (Cat# 163–20145, FUJIFILM Wako, Japan) prepared in 0.1 M phosphate buffer (pH 7.4). After rinsing three times in PBS (pH 7.4), the samples were submerged sequentially in 15 % and 30 % sucrose solutions, embedded in OCT compound (Cat# IA018, Sakura, CA, USA), and snap-frozen in liquid nitrogen. Ten-micrometer sections were cut using a CryoStar NX70 Cryostat (Expredia, MI, USA).

Sections were blocked for 1 h at room temperature in 10 % BSA and 0.1 % Triton X-100 (Cat# T8787, Sigma Aldrich, MO, USA) in PBS. Rabbit anti-insulin (1:1000, Cat# ab181547, RRID: AB_2716761, Abcam, AUS) and mIgG1 anti-glucagon (1:1000, Cat# ab10988, RRID: AB_297642, Abcam) antibodies were applied overnight at 4 °C in 1 % BSA and 0.1 % Triton X-100 in PBS. Sections were then incubated for 2 h with Alexa Fluor 488 goat anti-rabbit (1:500, Cat# A11034, Invitrogen, CA, USA) and Alexa Fluor 555 goat anti-mouse IgG1 (1:1000, Cat# A21127, Invitrogen), followed by DAPI (1:25000, Cat# D1306, Thermo Fisher, VIC, AUS) for 10 min. ProLong Gold Antifade Medium (Cat# P36962, Invitrogen) was used for mounting. Immunostaining and imaging parameters were standardised across genotypes and sections, with investigators blinded to genotype. Insulin- and glucagon-positive areas were quantified in 30 pancreatic islets per sample.

### 2.9 Statistical analysis

All analyses were performed using GraphPad Prism 9.5.1, with no exclusion criteria applied to the experimental data. The normality of datasets was assessed using the Shapiro–Wilk test, and all datasets passed. Data from indirect calorimetry were analysed using Student's t-tests, conducted separately for each age group. Time-course measurements of blood glucose and hormone levels during ipGTT and ipITT were evaluated using two-way ANOVA followed by Bonferroni multiple comparisons. The area under the curve (AUC) was calculated for individual mice using the trapezoid rule [22,23]. AUC values, baseline hormone and glycogen concentrations, and immunoreactive area data were also analysed with Student's t-tests, separately by age. All results are presented as mean ± SEM, and differences were deemed statistically significant at P < 0.05.

## 3 Results

### 3.1 Increased daily energy expenditure and food intake is observed in early symptomatic TDP-43^Q331K^ mice

**Fig. 1:**
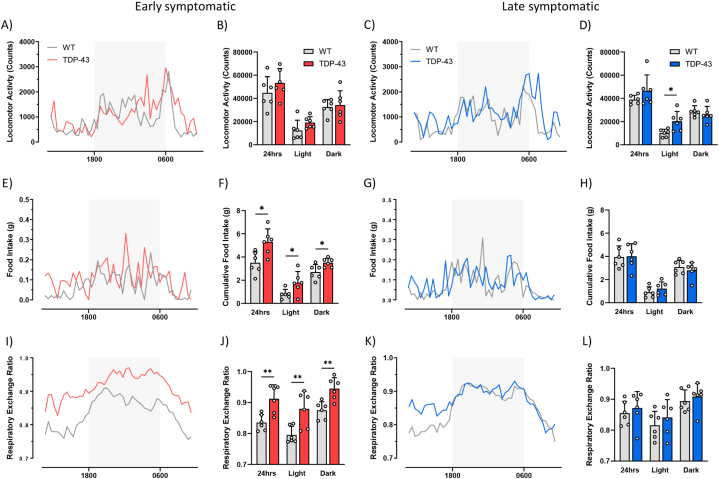
TDP-43^Q331K^ mice transiently increase food intake in the early stages of disease, potentially to compensate for the increase in daily energy expenditure (DEE).Indirect calorimetry analysis was performed on 40 weeks (early symptomatic) and 80 weeks of age (late symptomatic) mice using the Phenomaster TSE Metabolic Cage System. **(A**–**D)** The locomotor activity profile was assessed in WT (grey) and TDP-43^Q331K^ (red) mice at early symptomatic **(A)** and WT (grey) and TDP-43^Q331K^ (blue) mice at late symptomatic stage **(C)**. The cumulative locomotor activities were calculated for the total 24hr period and the light and dark phases at early symptomatic **(B)** and late symptomatic stage **(D)**. **(E**–**H)** The average food intake profile was measured at early symptomatic **(E)** and late symptomatic stage **(G)**. The cumulative food intake was calculated over the 24hr period as well as the light and dark phases at early symptomatic **(F)** and late symptomatic stage **(H)**. **(I**–**L)** The average respiratory exchange ratio (RER) profile was measured at early symptomatic **(I)** and late symptomatic stage **(K)**. The average RER was calculated over the 24hr period as well as the light and dark phases at early symptomatic **(J)** and late symptomatic stage **(L)**. All data presented as mean ± SEM; *n=6* for all groups. All bar graphs analysed by a Student's t-test; *∗p* < 0.05, *∗∗p* < 0.01.

Using indirect calorimetry, we measured food intake, locomotor activity and energy expenditure in mice throughout the progression of disease. We observed a progressive loss in both total body weight and lean mass in the TDP-43^Q331K^ mice compared to the WT controls (Table 1). Specifically, total body weight was reduced by 12 % (p = 0.002) in the early symptomatic (40 weeks of age) and 18 % (p = 0.032) in the late symptomatic (80 weeks of age) mice. Lean mass was also reduced by 8 % (p = 0.005) in the early symptomatic and 15 % (p = 0.010) in the late symptomatic TDP-43^Q331K^ mice compared to the WT controls. Daily energy expenditure (DEE) was not significantly altered between the WT and TDP-43^Q331K^ mice at either stage of disease. However, when we adjusted the DEE for both total body weight and lean mass using regression analysis, we found that the adjusted DEE was elevated at both stages (Table 1). When adjusted for total body weight, the DEE was increased by 1.9 kcal/day (p = 0.040) and 1.4 kcal/day (p = 0.025) in the TDP-43^Q331K^ mice at the early and late symptomatic stages, respectively. When adjusted for lean mass, the DEE in the TDP-43^Q331K^ mice was increased by 2.6 kcal/day (p = 0.005) and 1.5 kcal/day (p = 0.032) at the early and late symptomatic stages in comparison to the WT mice, respectively. Overall, this demonstrates that at any given weight or lean mass TDP-43^Q331K^ mice will expend more energy per day, throughout disease progression. Surprisingly, this increase in energy expenditure does not affect fat mass at these ages, with similar levels in fat mass observed in both genotypes at both early and late symptomatic stages of disease (Table 1). However, in a separate cohort of mice where body composition was measured at the end stage of disease (∼95 weeks of age), fat mass was reduced by 44 % in TDP-43^Q331K^ mice in comparison to the WT controls (p = 0.050). This was coupled with a 17 % and 24 % loss in lean mass (p = 0.020) and total body weight (p = 0.009), respectively. Furthermore, this increase in DEE appears to be independent of an increase in activity. Although we observed a 200 % increase in locomotor activity during the light phase in late symptomatic TDP-43^Q331K^ mice, similar levels of overall daily locomotor activity were observed between genotypes at both stages of disease (Fig. 1A–D).

**Table 1:** DEE expenditure is increased in TDP-43^Q331K^ mice when adjusted for weight.

Age	40 weeks (n = 6)		80 weeks (n = 6)		95 weeks (n = 3–5)	
Genotype	WT	TDP-43	WT	TDP-43	WT	TDP-43
**Total body weight (g)**	35.8 ± 0.8	31.8 ± 0.5 ∗∗	41.6 ± 2.6	34.2 ± 1.5 ∗	41.3 ± 3.6	31.3 ± 1.4 ∗∗
**Lean mass (g)**	27.7 ± 0.5	25.5 ± 0.3 ∗∗	29.6 ± 1.1	25.1 ± 0.8 ∗	30.7 ± 2.4	25.4 ± 1.1 ∗
**Fat mass (g)**	8.1 ± 1.9	6.4 ± 1.0	12.3 ± 1.7	9.1 ± 1.2	10.5 ± 1.5	5.9 ± 0.3 ∗
**DEE (kcal/day)**	8.8 ± 0.4	9.9 ± 0.4	9.0 ± 0.5	9.3 ± 0.3	–	–
**DEE adjusted to body weight (kcal/day)**	8.4 ± 0.6	10.3 ± 0.6 ∗	8.5 ± 0.3	9.9 ± 0.3 ∗	–	–
**DEE adjusted to lean mass (kcal/day)**	8.0 ± 0.4	10.6 ± 0.4 ∗∗	8.4 ± 0.4	9.9 ± 0.4 ∗	–	–

Data presented as mean ± SEM.

*∗p* < 0.05.

∗*∗p* < 0.01.

At the early symptomatic stage, TDP-43^Q331K^ mice were observed to consume 151 % more food (p = 0.013) in a 24-h period when compared to the WT mice (Fig. 1E and F). Moreover, TDP-43^Q331K^ mice consumed 224 % more food (p = 0.042) during the light phase and 130 % more food (p = 0.024) in the dark phase when compared to the WT mice. In contrast, at the late symptomatic stage, food intake was similar between genotypes (Fig. 1G and H). This suggests that increased food intake is specific to the early phase of the disease, potentially serving as an early compensatory mechanism to counterbalance the increase in energy expenditure associated with disease progression.

Additionally, the average daily respiratory exchange ratio (RER) was higher in early symptomatic TDP-43^Q331K^ mice compared to WT mice (p = 0.005, Fig. 1I and J). Further analysis during the light and dark phases showed that the average RER remained significantly higher in the TDP-43^Q331K^ mice during the light (p = 0.009) and dark phase (p = 0.004). This observed increase in RER is likely a result of the increased food intake in the TDP-43^Q331K^ mice, leading to a greater carbohydrate availability during both the light and dark phases. However, in the late symptomatic group, there were no significant differences in average RER across the time points (Fig. 1K and L). This further supports the notion that increases in food intake directly contribute to the increased RER observed in early symptomatic TDP-43^Q331K^ mice. No differences were observed in food intake or RER in TDP-43^WT^ mice compared to WT (Fig. S1), suggesting that these differences were a result of the Q331K mutation.

### 3.2 TDP-43^Q331K^ mice have increased insulin-independent glucose uptake during the later phases of disease progression

**Fig. 2:**
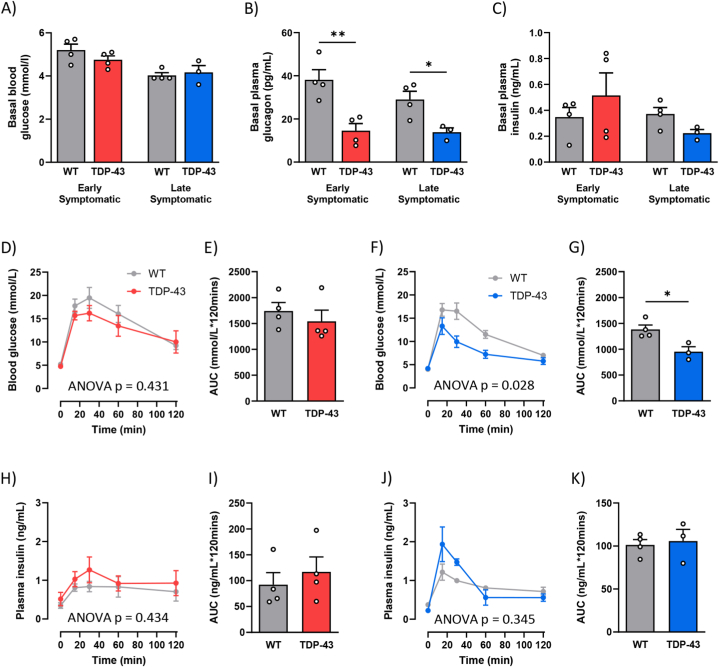
Basal blood glucose levels are maintained in TDP-43^Q331K^ mice, despite a loss of circulating plasma glucagon levels. Glucose uptake is increased in TDP-43^Q331K^ mice during the later stages of disease. **(A)** Basal blood glucose concentrations measured from tail bleed in 16hr fasted WT (grey) and TDP-43^Q331K^ (red) mice at 40 weeks (early symptomatic) and WT (grey) and TDP-43^Q331K^ (blue) mice at 80 weeks of age (late symptomatic). **(B)** Basal plasma glucagon concentrations measured from tail bleed in 16hr fasted WT (grey) and TDP-43^Q331K^ mice at early symptomatic (red) and late symptomatic stage (blue). **(C)** Basal plasma insulin concentrations measured from tail bleed in 16hr fasted WT (grey) and TDP-43^Q331K^ mice at early symptomatic (red) and late symptomatic stage (blue). **(D)** Time course of blood glucose concentrations during a glucose tolerance test (ipGTT) following a 2 g/kg intraperitoneal injection of glucose at the early symptomatic stage. **(E)** The average area under the curve (AUC) calculated from the blood glucose time course at the early symptomatic stage. **(F)** Time course of blood glucose concentrations during an ipGTT at the late symptomatic stage. **(G)** The average AUC calculated from the blood glucose time course at the late symptomatic stage. **(H)** Time course of insulin concentrations measured in plasma collected from tail bleeds throughout ipGTT in WT and TDP-43^Q331K^ mice at the early symptomatic stage. **(I)** The average AUC was calculated from the insulin time course as a measure of glucose-stimulated insulin release. **(J)** Time course of insulin concentrations measured in plasma collected from tail bleeds throughout ipGTT in WT and TDP-43^Q331K^ mice at the late symptomatic stage. **(K)** The average AUC was calculated from the insulin time course as a measure of glucose-stimulated insulin release. All data presented as mean ± SEM; *n=3-4* for all groups. Two-way ANOVA results listed on time course in panels **D**, **F**, **H** and **J** is the overall significance between genotypes, All bar graphs analysed by a Student's t-test; *∗p* < 0.05, *∗∗p* < 0.01.

We next aimed to confirm if glucose handling was changed in the TDP-43^Q331K^ mice by using ipGTT. Initially, we observed no changes in basal blood glucose levels between the genotypes at either age, following a 16-h fast (Fig. 2A). However, there was a significant reduction of 62 % and 52 % in plasma glucagon levels in early (p = 0.0064) and late (p = 0.0255) symptomatic TDP-43^Q331K^ mice compared to the WT groups, respectively (Fig. 2B). Similar levels of plasma insulin were found between the WT and TDP-43^Q331K^ mice at either stage of disease (Fig. 2C). Taken together, this suggests that an alternate mechanism might be regulating blood glucose concentrations during fasting. No differences were found in basal blood glucose or hormone levels between TDP-43^WT^ and WT mice (Fig. S2). At the onset of motor symptoms, both WT and TDP-43^Q331K^ mice exhibited a similar response to exogenous glucose (Fig. 2D and E). However, late symptomatic TDP-43^Q331K^ mice appeared to be more glucose tolerant, demonstrating a faster rate of blood glucose clearance evidenced by a 31 % reduction in AUC (p = 0.0204, Fig. 2F and G) compared to WT mice. Interestingly, this increased glucose uptake in TDP-43^Q331K^ mice at 80 weeks of age appeared to be independent of insulin, as no significant changes were observed in glucose-stimulated plasma insulin concentrations at either age (Fig. 2H–K). No changes in glucose handling were observed in TDP-43^WT^ mice (Fig. S2). These findings indicate that glucose handling is altered throughout disease progression, resulting in an increase in insulin-independent glucose uptake in later stages of disease in TDP-43^Q331K^ mice.

### 3.3 TDP-43^Q331K^ mice are insulin tolerant

**Fig. 3:**
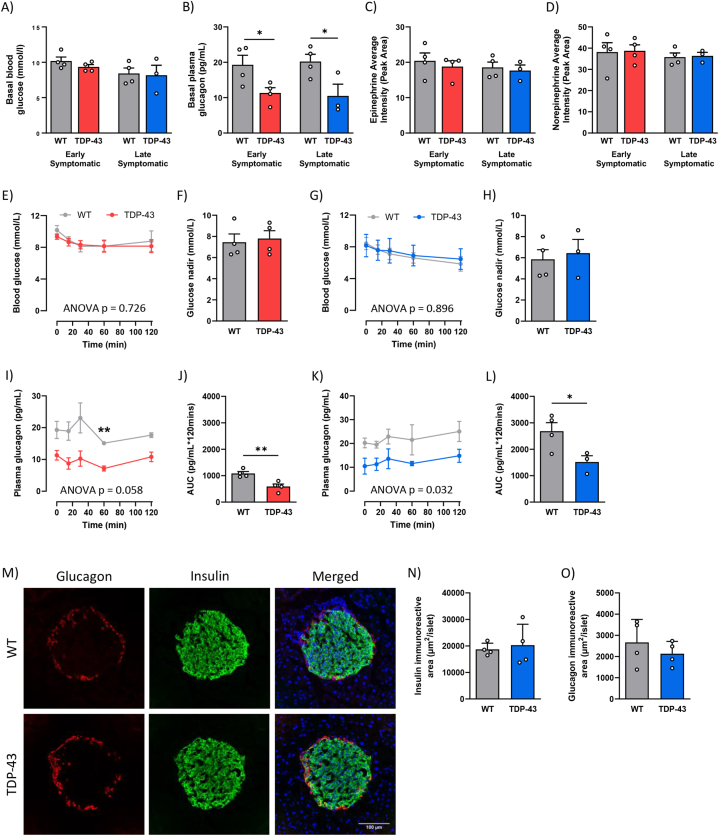
TDP-43^Q331K^ mice are insulin tolerant throughout disease progression. **(A)** Basal blood glucose concentrations measured from tail bleed in 6hr fasted WT (grey) and TDP-43^Q331K^ mice at 40 weeks (early symptomatic; red) and 80 weeks of age (late symptomatic; blue). **(B)** Basal plasma glucagon concentrations measured from tail bleed in 6hr fasted WT (grey) and TDP-43^Q331K^ mice at early symptomatic (red) and late symptomatic stage (blue). **(C)** Basal plasma epinephrine concentrations measured from tail bleed in 6hr fasted WT (grey) and TDP-43^Q331K^ mice at early symptomatic (red) and late symptomatic stage (blue). **(D)** Basal plasma norepinephrine concentrations measured from tail bleed in 6hr fasted WT (grey) and TDP-43^Q331K^ mice at early symptomatic (red) and late symptomatic stage (blue). **(E)** Time course of blood glucose concentrations during an insulin tolerance test (ipITT) following a 0.5 IU/kg intraperitoneal injection of insulin at the early symptomatic stage. **(F)** The average area under the curve (AUC) calculated from the blood glucose time course at the early symptomatic stage. **(G)** Time course of blood glucose concentrations during an ipITT at the late symptomatic stage. **(H)** The average AUC calculated from the blood glucose time course at late symptomatic stage. **(I)** Time course of glucagon concentrations measured in plasma collected from tail bleeds throughout ipITT in WT and TDP-43^Q331K^ mice at early symptomatic stage. **(J)** The average AUC was calculated from the glucagon time course at early symptomatic stage. **(K)** Time course of glucagon concentrations measured in plasma collected from tail bleeds throughout ipITT at late symptomatic stage. **(L)** The average AUC was calculated from the glucagon time course at late symptomatic stage. **(M)** Representative images of glucagon-positive α-cells (red) and insulin-positive β-cells (green) in the pancreas of WT and TDP-43^Q331K^ mice at late symptomatic stage. **(N)** The average immunoreactive area of insulin-positive β-cells in the pancreatic islet cells calculated from 30 islets per animal. **(O)** The average immunoreactive area of glucagon-positive α-cells in the pancreatic islet cells from 30 islets per animal. All data presented as mean ± SEM; *n=3-4* for all groups. Two-way ANOVA results listed on time course in panels **E**, **G, I** and **K** is the overall significance between genotypes, Bonferroni post-test was used to determine significant changes at specific time points. All bar graphs analysed by a Student's t-test; *∗p* < 0.05, *∗∗p* < 0.01.

To evaluate insulin tolerance in TDP-43^Q331K^ mice, we conducted an ipITT following a 6-h fast. At both the early and late stages of disease there were no differences in basal blood glucose concentrations between genotypes (Fig. 3A). However, a similar trend in plasma glucagon concentrations was observed in this shorter fasting period, consistent with the results after the 16-h fast (Fig. 3B). Plasma glucagon concentrations were reduced by 41 % in early symptomatic TDP-43^Q331K^ mice compared to WT mice (p = 0.042). Similarly, there was a 48 % reduction in baseline plasma glucagon concentrations between the late symptomatic TDP-43^Q331K^ and WT groups (p = 0.046). No differences were observed in the fasted plasma epinephrine or norepinephrine levels at either stage of disease, suggesting that this pathway does not compensate for the glucagon deficiency in the TDP43^Q331K^ mice (Fig. 3C and D). This data further confirms that an alternate mechanism is involved in maintaining blood glucose concentrations during fasting periods. Furthermore, the TDP-43^WT^ mice were found to have similar glucagon concentrations as WT mice (Fig. S3), further indicating that this loss in glucagon is specific to the Q331K mutation.

At both disease stages, both genotypes showed a similar response to exogenous human insulin, as evidenced by the similar blood glucose nadir during the ITT (Fig. 3E–H). No differences were found in the TDP-43^WT^ mice as well (Fig. S3), suggesting that neither WT nor the Q331K mutated TDP-43 induces insulin resistance in these mice. During the ipITT, glucagon concentrations are expected to increase in response to the drop in blood glucose concentrations. When we measured glucagon concentrations in plasma collected throughout the ipITT, we saw a similar pattern in the change in circulating glucagon concentrations over time. However, due to the lower basal levels the overall AUC was significantly lower in the TDP-43^Q331K^ mice (Figure I–L). Specifically, we found that the AUC was 46 % and 43 % lower at the early symptomatic (p = 0.006) and late symptomatic (p = 0.043) stages of disease, respectively. This suggests that glucagon secretion may be altered in the TDP-43^Q331K^ mice. No differences were observed between the WT and TDP-43^WT^ mice, further indicating that these changes are specific to the Q331K mutation.

To investigate whether the reduction in glucagon secretion was caused by changes in the pancreatic islets, we measured the area of glucagon-positive α-cells and insulin-positive β-cells in pancreatic cryosections from mice at the late symptomatic stage. Similarly, no significant changes were found in the average area of α-cells (p = 0.420) and β-cells (p = 0.755) in the pancreas between the TDP-43^Q331K^ and WT mice at this age (Fig. 3M−O). This indicates that there are no structural changes in the pancreatic islets that might be responsible for the reduction in circulating glucagon concentrations.

### 3.4 Glucagon signalling is unaltered in TDP-43^Q331K^ mice

**Fig. 4:**
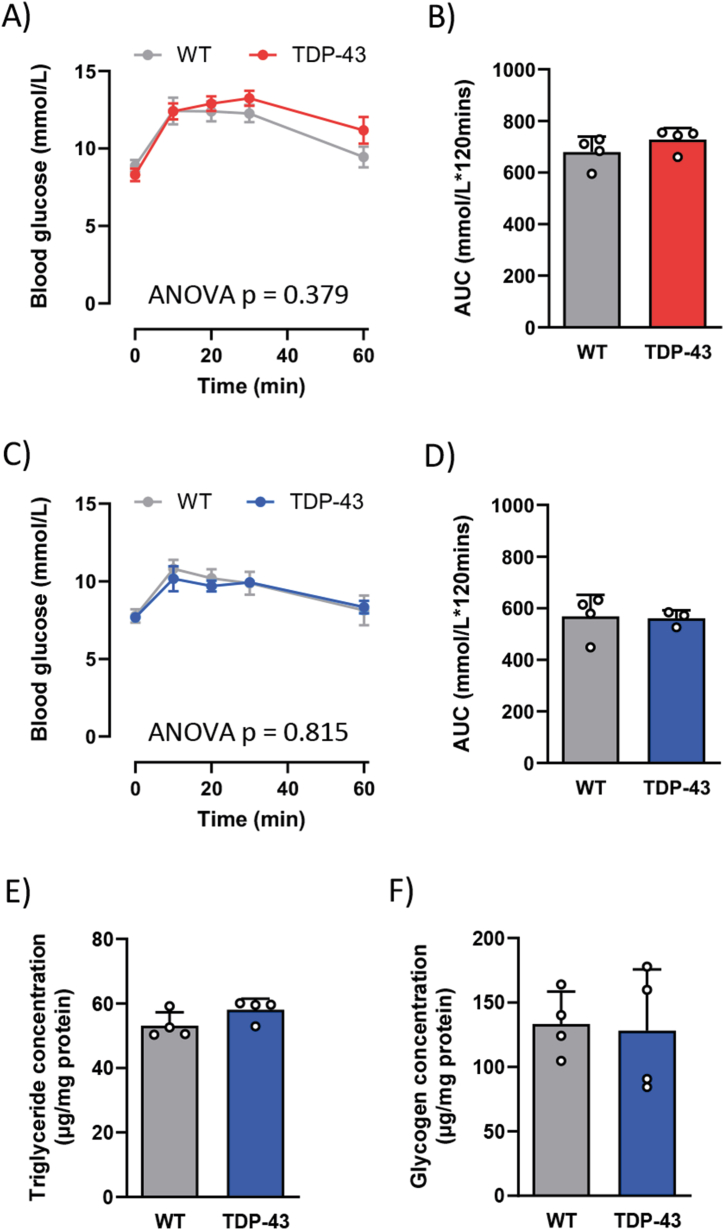
Glucagon tolerance is unchanged in TDP-43^Q331K^ mice throughout disease progression. **(A**) Time course of blood glucose concentrations during a glucagon challenge following a 250 μg/kg intraperitoneal injection of glucagon in WT (grey) and TDP-43^Q331K^ (red) mice at 40 weeks of age (early symptomatic stage). **(B)** The average area under the curve (AUC) calculated from the blood glucose time course at the early symptomatic stage. **(C)** Time course of blood glucose concentrations during a following a glucagon challenge in WT (grey) and TDP-43^Q331K^ (blue) mice at 80 weeks of age (late symptomatic stage). **(D)** The average AUC calculated from the blood glucose time course at the late symptomatic stage. **(E)** Triglyceride concentrations from liver homogenates collected from WT (grey) and TDP-43^Q331K^ (blue) mice at the late symptomatic stage. **(F)** Glycogen concentrations from liver homogenates collected from WT (grey) and TDP-43^Q331K^ (blue) mice at the late symptomatic stage. All data presented as mean ± SEM; *n=3-4* for all groups. Two-way ANOVA results listed on time course in panels **A** and **C** is the overall significance between genotypes, Bonferroni post-test was used to determine significant changes at specific time points. All bar graphs analysed by a Student's t-test.

After observing reduced glucagon concentrations in fasted TDP-43^Q331K^ mice, an *in vivo* glucagon challenge was conducted to assess glucagon signalling. Surprisingly, the change in blood glucose concentrations in response to exogenous glucagon was similar between genotypes at both ages (Fig. 4A–D). This suggests that glucagon signalling remains intact in TDP-43^Q331K^ mice throughout disease progression. As glucagon signalling plays a role in glycogenolysis and triglyceride metabolism, the concentration of glycogen and triglycerides in the liver of late symptomatic mice was measured. We found that both the triglyceride and glycogen levels were similar between genotypes at this age (Fig. 4E and F). These findings further support the notion that, despite the loss in glucagon concentrations, glucagon signalling within the liver remains functional.

## 4 Discussion

The major finding of this current study is that TDP-43^Q331K^ mice have significantly increased DEE starting from the early symptomatic stages of disease. This increased DEE coincides with a transient increase in food intake, potentially explaining the lack of changes in fat mass in these mice. Furthermore, during the later stages of the disease, TDP-43^Q331K^ mice demonstrate enhanced glucose clearance, independent of insulin, as evidenced by stable circulating insulin concentrations during the ipGTT. Notably, at both disease stages, TDP-43^Q331K^ mice demonstrated no evidence of insulin or glucagon intolerance, despite reduced glucagon concentrations observed during both short and prolonged fasting periods. Additionally, epinephrine and norepinephrine levels were unchanged throughout disease progression in TDP-43^Q331K^ mice. These findings suggests that alternate mechanisms exist for maintaining liver glycogen and triglyceride levels, as well as blood glucose levels during fasting in TDP-43^Q331K^ mice. Importantly, we also found that increased expression of wild-type human TDP-43 had minimal impact on the metabolic profile in this mouse model.

The reduction in lean mass observed in TDP-43^Q331K^ mice during both early and late symptomatic stages of disease aligns with findings from various ALS rodent models [17]. Employing a regression-based analysis to adjust for both total body mass and lean mass, we determined that DEE remained increased at both disease stages. This analytical approach is crucial for discerning mass-dependent and -independent effects on metabolic rates [24], revealing that TDP-43^Q331K^ mice have an elevated metabolic rate relative to WT mice, regardless of their mass. While previous studies on TDP-43^A315T^ mice have reported no change in energy expenditure [13], the observed increase in DEE in TDP-43^Q331K^ mice is consistent with several studies demonstrating heightened metabolic rates in patients with ALS [[25], [26], [27]]. Notably, this increase in energy expenditure in patients correlates with weight loss and poorer disease outcomes [25,27]. Interestingly, although we did not observe a loss of fat mass in TDP-43^Q331K^ mice at either age, likely due to the increased food intake during the early onset stage, fat mass was significantly reduced at the later stage (∼95 weeks of age). Analysis of feeding behaviour revealed that TDP-43^Q331K^ mice exhibit a transient increase in food intake during early symptomatic stages, which may contribute to maintaining fat mass despite elevated energy expenditure. However, this compensatory behaviour is insufficient in later disease stages, potentially leading to fat mass reduction as an alternate fuel source to compensate for increased energy expenditure. These findings highlight the interplay between feeding behaviour and energy homeostasis in ALS progression. Moreover, increased energy expenditure and changes in body composition observed in ALS models may also be influenced by alterations in leptin signalling. Leptin, an adipokine regulating energy balance and appetite, is often linked with fat mass and metabolic rate. Although we did not directly assess leptin expression in this study, previous research has shown that ALS patients and rodent models can exhibit altered leptin levels, which may impact disease progression and energy regulation [[28], [29], [30]]. Future studies examining leptin expression in TDP-43^Q331K^ mice could help clarify its role in mediating the metabolic shifts seen with disease progression.

Subsequently, we investigated the potential contribution of changes in glucose and insulin tolerance to the observed increase in energy expenditure. We observed that blood glucose clearance was more efficient in TDP-43^Q331K^ mice at later stages of disease. This finding is consistent with previous reports, including our own study, where glucose clearance was increased in mid-symptomatic SOD1^G93A^ mice [17]. Similarly, elevated glucose clearance was reported in SOD1^G86R^ mice, with increased 2-deoxyglucose uptake observed in white adipose tissue [28]. However, it is important to note that conflicting results have been reported in other studies, showing either no change or even a loss of glucose clearance throughout disease progression in rodent models of ALS [13,16,31]. In patients with ALS, glucose uptake was found to be increased in denervated forearm and skeletal muscle but decreased in the cerebral cortex and spinal cord [[32], [33], [34], [35]]. These findings suggest that whole-body glucose clearance may not accurately reflect changes in individual tissues. Such inconsistencies underscore the need for further research to fully understand the underlying mechanisms driving alterations in glucose clearance in the context of ALS. Additionally, mitochondrial dysfunction could contribute to the metabolic changes observed in TDP-43^Q331K^ mice. Mitochondria play a key role in energy production, and impaired mitochondrial function has been implicated in ALS pathogenesis. Alterations in mitochondrial bioenergetics, including changes in ATP production and reactive oxygen species generation, may affect metabolic processes such as glucose uptake and energy expenditure. Future studies investigating mitochondrial function in this model could provide valuable insights into how mitochondrial dysfunction contributes to the observed metabolic alterations and ALS progression.

In contrast to the results of the ipGTT, our findings from the ipITT indicate that insulin sensitivity is unaltered in TDP-43^Q331K^ mice. This corroborates our findings in the SOD1^G93A^ mouse model of ALS, which also showed no signs of insulin resistance throughout disease progression [17]. However, varying results have been reported in ALS patients. While one study found that insulin sensitivity remained unchanged during a euglycemic insulin clamp [36], another study showed that ALS patients required a significantly lower glucose infusion rate to maintain glycemia, indicating that the patients in this study were insulin resistant [14]. Additional evidence supporting potential insulin resistance in ALS comes from a study using mice overexpressing both WT and A315-mutated TDP-43 in skeletal muscle. These mice exhibited impaired insulin mediated GLUT4 translocation to cell membrane in the flexor digitorum brevis muscle, leading to reduced uptake of a fluorescently tagged glucose analogue in response to insulin. This suggests that these mice may indeed be insulin resistant. One limitation of this research was our inability to identify the precise tissues responsible for glucose uptake when exposed to exogenous insulin. Furthermore, it must also be noted that both wild-type and mutated TDP-43 expression in our mouse model was restricted to the CNS, with minimal expression in peripheral tissues. Therefore, it would be of interest to determine if global TDP-43^Q331K^ expression alters insulin resistance in these mice.

Overall, our findings suggest that while insulin-mediated glucose disposal remains intact in TDP-43^Q331K^ mice, there is an increase in insulin-independent glucose uptake at the later stages of the disease. This indicates that insulin signalling remains functional in these mice, while other mechanisms may be driving the enhanced glucose uptake during disease progression. Interestingly, some of the metabolic changes observed in TDP-43^Q331K^ mice, particularly alterations in glucose tolerance and hormone secretion, resemble features commonly seen in type 2 diabetes mellitus. While our data do not confirm a canonical type 2 diabetes mellitus profile in these mice, the observed parallels raise the question of whether antidiabetic agents could play a role in modulating ALS progression. Recent studies have discussed the potential of antidiabetic medications, such as metformin, pioglitazone, and GLP-1 receptor agonists, to target metabolic pathways in ALS [37]. Additionally, large-scale epidemiological and mechanistic studies suggest a link between type 2 diabetes mellitus and ALS, with connections varying by age and disease stage, emphasising the need to investigate the metabolic mechanisms underlying each condition [38]. Future studies involving global TDP-43^Q331K^ expression, as well as testing the efficacy of antidiabetic drugs in this model, could reveal whether modulating type 2 diabetes mellitus-related pathways influences ALS onset or progression.

Regarding gastrointestinal (GI) pathology, we acknowledge the possibility that GI dysfunction could contribute to the metabolic changes observed in TDP-43^Q331K^ mice. While we did not observe overt signs of GI distress, such as changes in stool consistency or feeding behaviour, we cannot rule out the possibility that subtle GI impairments may influence energy balance. GI pathology, including impaired nutrient absorption or digestion, is often associated with neurodegenerative diseases and could contribute to altered metabolism. Future studies incorporating more focused GI assessments could help clarify whether GI dysfunction plays a role in the metabolic alterations seen in this model.

To investigate the elements influencing the changes in glucose metabolism during the late symptomatic phase, we evaluated the levels of the crucial glucoregulatory hormones, insulin and glucagon. Our findings revealed no significant changes in insulin secretion in TDP-43^Q331K^ mice throughout disease progression, supporting the notion that the increase in glucose disposal is likely driven by an insulin-independent mechanism. However, these results conflict with previous studies that have shown a reduced insulin response following a GTT in both ALS patients and mouse models of disease [17,39]. In patients the impaired synthesis or release of insulin was attributed to damage to the pancreatic islet cells [39]. In our study, we found no change in the structure or average area of insulin-positive β-cells in TDP-43^Q331K^ mice. Interestingly, it is worth noting that nuclear localisation of TDP-43 was lost in islets of autopsied pancreata from ALS patients [40]. This loss of nuclear localisation was shown to inhibit insulin exocytosis by downregulating L-type voltage dependent calcium channels, thereby reducing first-phase insulin secretion in MIN6 cells [40]. Since our mouse model only expresses TDP-43 in the CNS, it would be of interest to investigate whether global expression of TDP-43^Q331K^ would affect TDP-43 nuclear localisation in the pancreatic islets or insulin secretion.

In contrast to circulating insulin concentrations, we found that plasma glucagon concentrations were reduced in TDP-43^Q331K^ mice under fasting conditions, throughout disease progression. These results contradict findings from both animal models and ALS patients. Previously, we demonstrated that glucagon levels are elevated in SOD1^G93A^ mice when fasted, yet despite this increase, the mice were unable to maintain blood glucose concentrations [17]. Similarly, in ALS patients, increased fasting and postprandial glucagon concentrations were coupled with reduced fasting glucose, indicating glucagon resistance in both patients and SOD1^G93A^ mice. Interestingly, TDP-43^Q331K^ mice were able to maintain their blood glucose levels despite the loss of circulating glucagon. Glucagon signalling in the liver plays a critical role in inducing glycogenolysis (the breakdown of glycogen into glucose) and inhibiting triglyceride storage. However, we found that liver glycogen concentrations were similar between TDP-43^Q331K^ and WT mice, despite the lack of enhanced glucagon signalling in the TDP-43^Q331K^ mice. Together, this suggests the involvement of an alternate mechanism in maintaining fasting glucose levels in these mice. We also investigated plasma concentrations of epinephrine and norepinephrine, two adrenal hormones known to alter blood glucose concentrations, controlling the release of glucagon and insulin from the pancreatic islets, and directly stimulating glycogenolysis in the liver [41]. However, we found no differences in the levels of these hormones between the TDP-43^Q331K^ and WT mice at either disease stage, indicating that fasting glucose homeostasis and pancreatic glucagon secretion in TDP-43^Q331K^ mice are not maintained by these hormones.

In addition, given the evidence that corticosterone can elevate blood glucose levels by increasing gluconeogenesis, glycogenolysis and triglyceride breakdown in the liver [42,43], it is possible that elevated corticosterone levels contribute to glucose homeostasis in TDP-43^Q331K^ mice. Altered hypothalamic-pituitary-adrenal axis activity has been reported in ALS [44], with elevated plasma cortisol levels associated with rapid disease progression in ALS patients [45]. Although we did not measure circulating corticosterone levels in this study, future investigations examining this hormone in TDP-43^Q331K^ mice would help clarify its potential role in maintaining fasting glucose levels. Glucagon secretion is regulated by an array of hormones produced in both the pancreas and hypothalamus. Therefore, further investigation is required to determine the mechanisms driving the loss in glucagon secretion and regulating fasting blood glucose homeostasis in this mouse model of ALS. Understanding these potential mechanisms could shed light on the intricate regulation of glucose homeostasis in ALS and offer new avenues for therapeutic exploration.

## 5 Conclusions

In summary, our study elucidates distinct metabolic effects associated with TDP-43 overexpression in ALS. While wild-type TDP-43 overexpression has minimal effect on glucose homeostasis and whole-body glucose metabolism, the expression of the mutated TDP-43^Q331K^ leads to increased metabolic rate throughout disease progression and a transient rise in food intake. Remarkably, our results also indicate that glucose uptake is increased in the later stages of disease, driven by insulin-independent mechanisms. Furthermore, we demonstrate that TDP-43^Q331K^ mice maintain fasting blood glucose levels despite reduced circulating glucagon levels, indicating alternative mechanisms regulating glucose metabolism in this specific mouse model of ALS. Notably, our study highlights the unique metabolic profile of TDP-43^Q331K^ mice compared to the well-studied SOD1^G93A^ mouse model of ALS. This underscores the complexity and heterogeneity of metabolic alterations observed in different ALS models and patients.

## CRediT authorship contribution statement

**Tanya S. McDonald:** Writing – review & editing, Writing – original draft, Investigation, Funding acquisition, Formal analysis, Conceptualization. **Cedric S. Cui:** Writing – review & editing, Investigation, Formal analysis. **Titaya Lerskiatiphanich:** Writing – review & editing, Investigation, Formal analysis. **Jianina Marallag:** Writing – review & editing, Investigation, Formal analysis. **John D. Lee:** Writing – review & editing, Supervision, Resources, Funding acquisition, Conceptualization.

## Declarations

The study was approved by the University of Queensland Animal Ethics Committee (199/17) and adhered to the policies and regulations governing animal experimentation. Experiments were conducted in accordance with the Queensland Government Animal Research Act 2001, the associated Animal Care and Protection Regulations (2002 and 2008), and the Australian Code of Practice for the Care and Use of Animals for Scientific Purposes, 8th Edition.

## Data availability statement

Data will be made available on request. For requesting data, please contact the corresponding author.

## Funding

This work was supported by Motor Neuron Disease Research Australia (Grant number IG2229).

## Declaration of competing interest

The authors declare the following financial interests/personal relationships which may be considered as potential competing interests: John Lee reports financial support was provided by 10.13039/100016887Motor Neurone Disease Research Australia. If there are other authors, they declare that they have no known competing financial interests or personal relationships that could have appeared to influence the work reported in this paper.
