# Supplementary material for: Metabolic rate and insulin-independent glucose uptake increase in a TDP-43Q331K mouse model of amyotrophic lateral sclerosis
Source: Heliyon. 2025 Feb 5;11(3):e42482. doi: 10.1016/j.heliyon.2025.e42482 (PMC11849610; doi:10.1016/j.heliyon.2025.e42482)
Supplement: Multimedia component 1 [file mmc1.docx]

**Supplementary Figures**


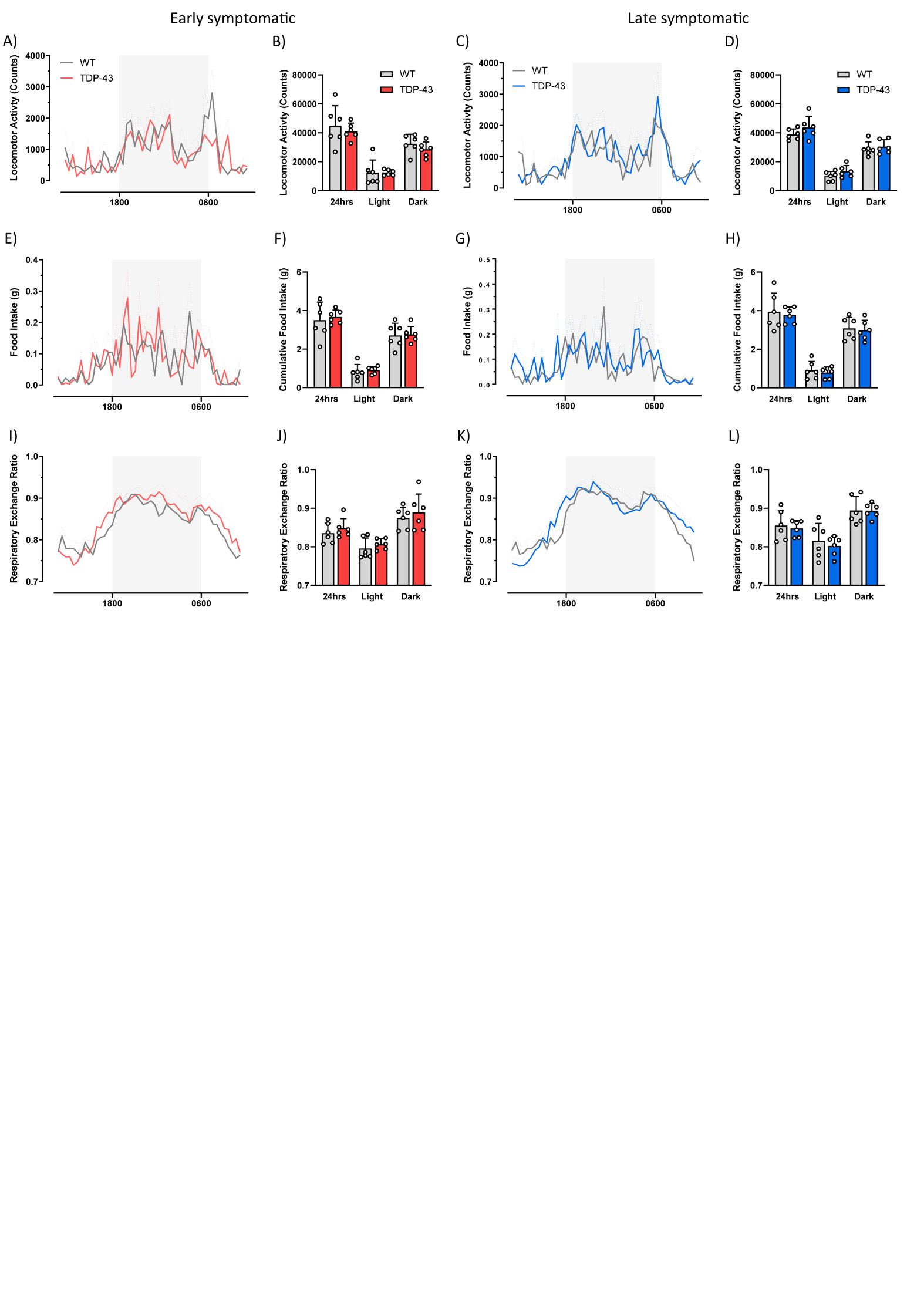


**Supplementary Figure 1. No differences were found in food intake or activity in TDP-43^WT^ mice throughout disease progression.**

Indirect calorimetry analysis was performed on 40 weeks (early symptomatic) and 80 weeks of age (late symptomatic) males using the Phenomaster TSE Metabolic Cage System. **(A-D)** The locomotor activity profile was assessed in WT (grey) and TDP-43^WT^ (red) mice at 40 weeks **(A)** and WT (grey) and TDP-43^WT^ (blue) mice at 80 weeks of age **(C)**. The cumulative locomotor activities were calculated for the total 24hr period and the light and dark phases at 40 weeks **(B)** and 80 weeks of age **(D). (E-H)** The average food intake profile was measured at 40 weeks **(E)** and 80 weeks of age **(G)**. The cumulative food intake was calculated over the 24hr period as well as the light and dark phases at 40 weeks **(F)** and 80 weeks of age **(H). (I-L)** The average respiratory exchange ratio (RER) profile was measured at 40 weeks **(I)** and 80 weeks of age **(K)**. The average RER was calculated over the 24hr period as well as the light and dark phases at 40 weeks **(J)** and 80 weeks of age **(L).** All data presented as mean ± SEM; *n=6* for all groups. All bar graphs analysed by a Student’s t-test.


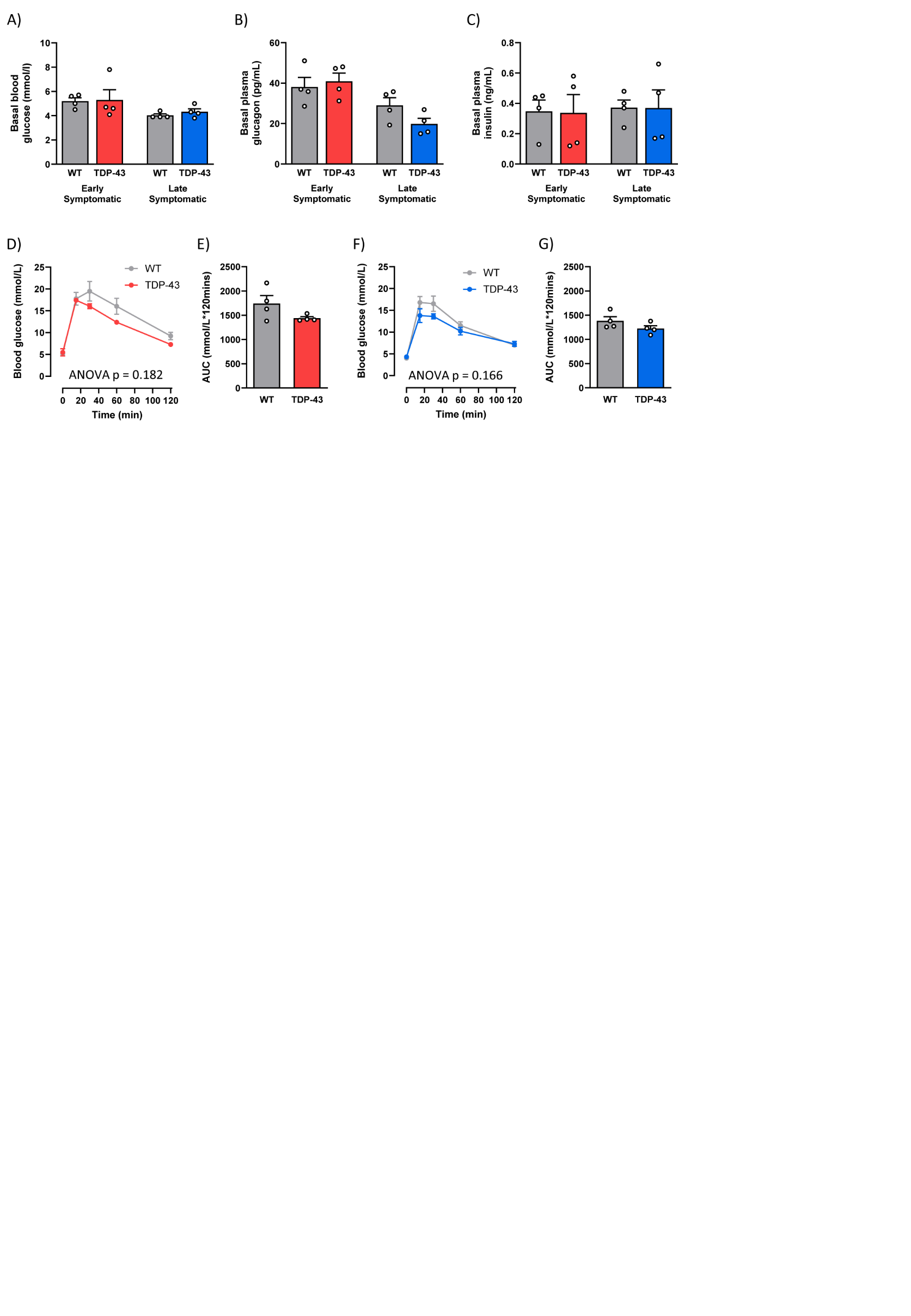


**Supplementary Figure 2. Basal blood glucose, plasma hormone levels and glucose tolerance were similar between WT and TDP-43^WT^ at both stages of disease.**

**(A)** Basal blood glucose concentrations measured from tail bleed in 16hr fasted WT (grey) and TDP-43^WT^ (red) mice at 40 weeks and WT (grey) and TDP-43^WT^ (blue) mice at 80 weeks of age. **(B)** Basal plasma glucagon concentrations measured from tail bleed in 16hr fasted WT (grey) and TDP-43^WT^ mice at 40 weeks (early symptomatic; red) and 80 weeks of age (late symptomatic; blue). **(C)** Basal plasma insulin concentrations measured from tail bleed in 16hr fasted WT (grey) and TDP-43^WT^ mice at 40 weeks (early symptomatic; red) and 80 weeks of age (late symptomatic; blue). **(D)** Time course of blood glucose concentrations during a glucose tolerance test (ipGTT) following a 2 g/kg intraperitoneal injection of glucose at the early symptomatic stage. **(E)** The average area under the curve (AUC) calculated from the blood glucose time course at the early symptomatic stage. **(F)** Time course of blood glucose concentrations during an ipGTT at the late symptomatic stage. **(G)** The average AUC calculated from the blood glucose time course at the late symptomatic stage. All data presented as mean ± SEM; *n=3-4* for all groups. Two-way ANOVA results listed on time course in panels **D** and **F** is the overall significance between genotypes. All bar graphs analysed by a Student’s t-test.


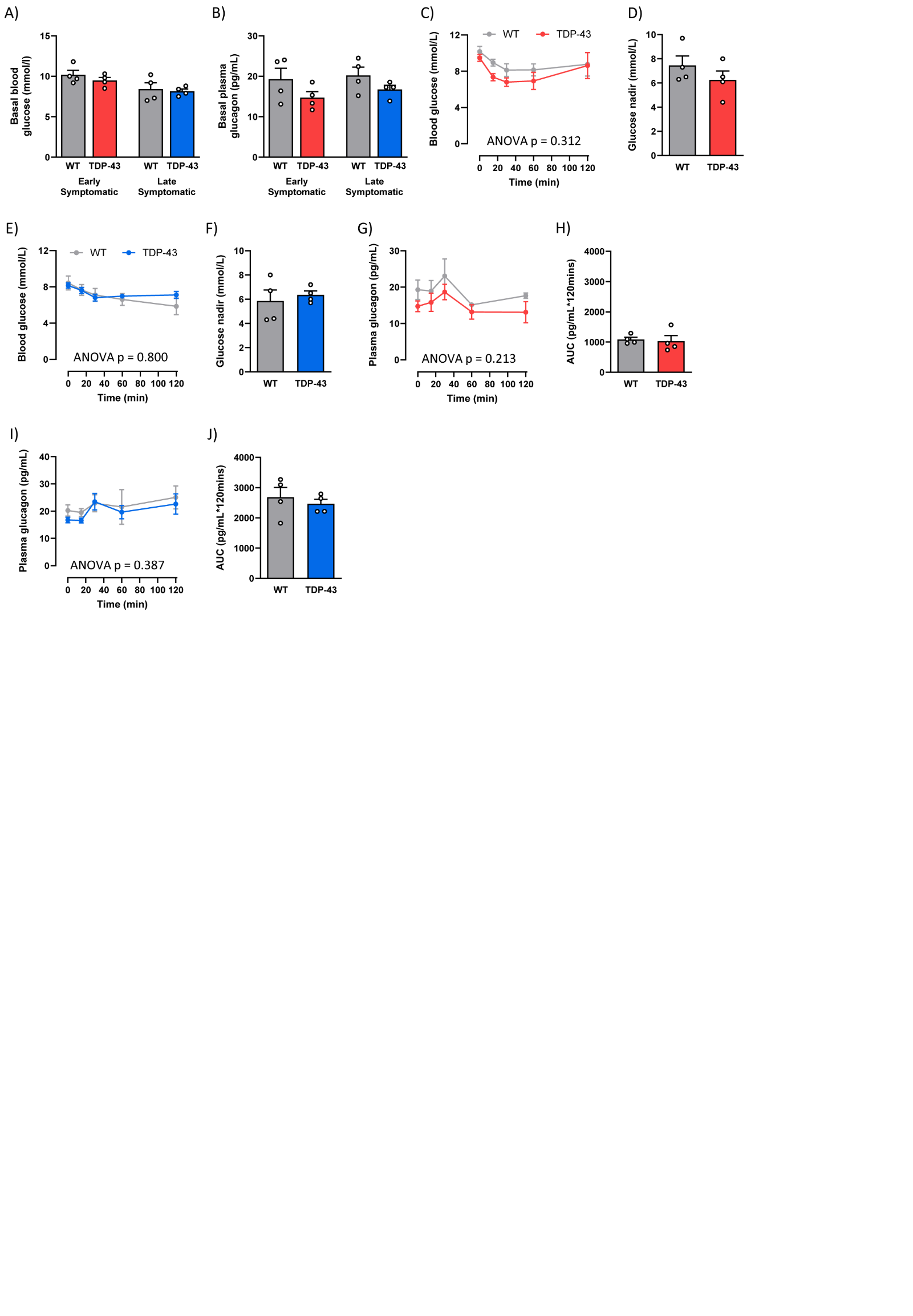


**Supplementary Figure 3. TDP-43^WT^ mice are insulin tolerant throughout disease progression.**

**(A)** Basal blood glucose concentrations measured from tail bleed in 6hr fasted WT (grey) and TDP-43^Q331K^ mice at 40 weeks (early symptomatic; red) and 80 weeks of age (late symptomatic; blue). **(B)** Basal plasma glucagon concentrations measured from tail bleed in 6hr fasted WT (grey) and TDP-43^Q331K^ mice at 40 weeks (early symptomatic; red) and 80 weeks of age (late symptomatic; blue). **(C)** Time course of blood glucose concentrations during an insulin tolerance test (ipITT) following a 0.5 IU/kg intraperitoneal injection of insulin at the early symptomatic stage. **(D)** The average area under the curve (AUC) calculated from the blood glucose time course at the early symptomatic stage. **(E)** Time course of blood glucose concentrations during an ipITT at the late symptomatic stage. **(F)** The average AUC calculated from the blood glucose time course at late symptomatic stage. **(G)** Time course of glucagon concentrations measured in plasma collected from tail bleeds throughout ipITT in WT and TDP-43^WT^ mice at early symptomatic stage**. (H)** The average AUC was calculated from the glucagon time course at early symptomatic stage. **(I)** Time course of glucagon concentrations measured in plasma collected from tail bleeds throughout ipITT at late symptomatic stage**. (J)** The average AUC was calculated from the glucagon time course at late symptomatic stage. All data presented as mean ± SEM; *n=3-4* for all groups. Two-way ANOVA results listed on time course in panels **C**, **E, G,** and **I** is the overall significance between genotypes, Bonferroni post-test was used to determine significant changes at specific time points. All bar graphs analysed by a Student’s t-test.


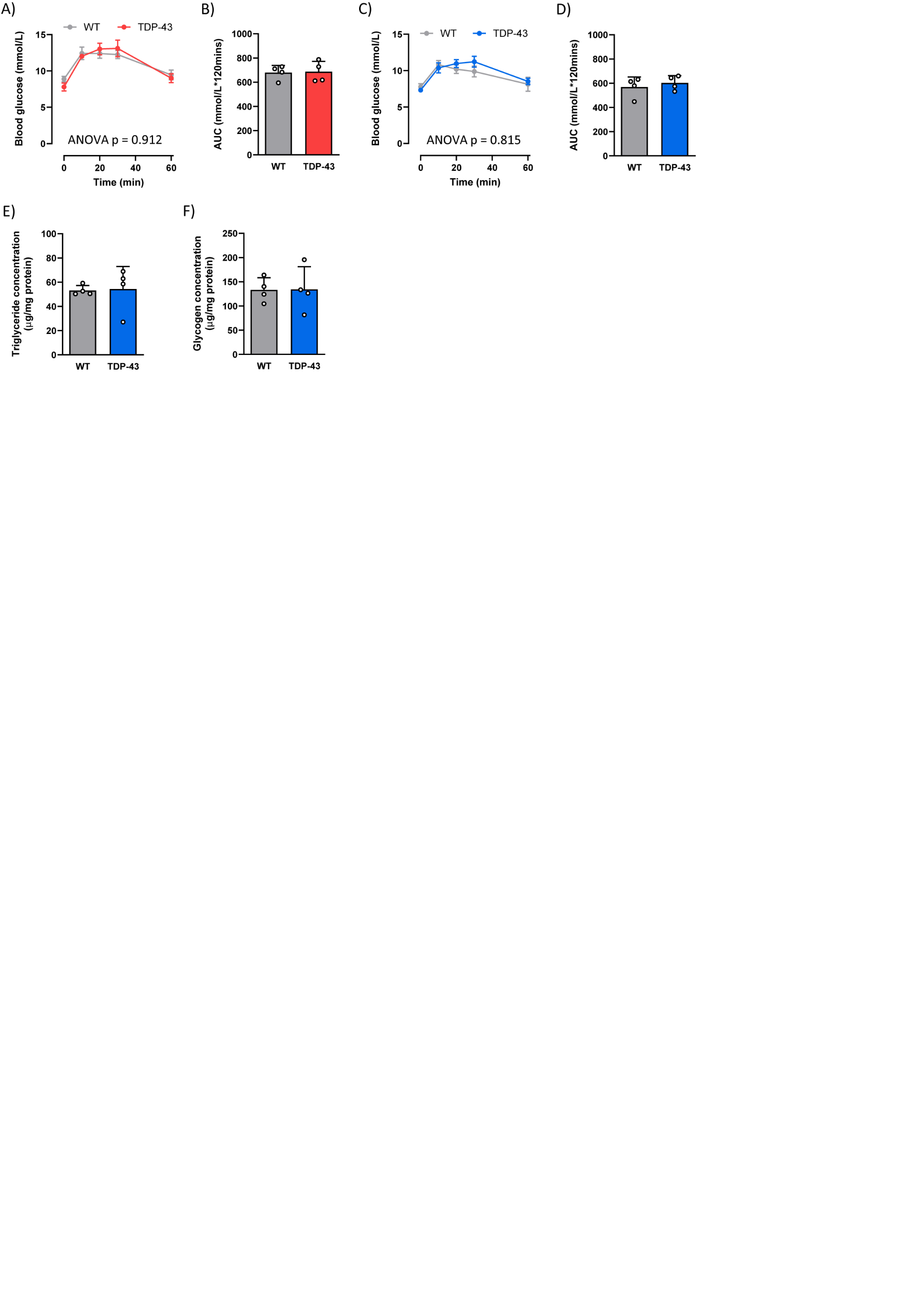


**Supplementary Figure 4. Glucagon tolerance is unchanged in TDP-43^WT^ mice throughout disease progression.**

**(A**) Time course of blood glucose concentrations during a glucagon challenge following a 250 µg/kg intraperitoneal injection of glucagon in WT (grey) and TDP-43^WT^ (red) mice at the early symptomatic stage. **(B)** The average area under the curve (AUC) calculated from the blood glucose time course at the early symptomatic stage. **(C)** Time course of blood glucose concentrations during a glucagon challenge in WT (grey) and TDP-43^Q331K^ (blue) mice at the late symptomatic stage. **(D)** The average AUC calculated from the blood glucose time course at the late symptomatic stage. **(E)** Triglyceride concentrations from liver homogenates collected from WT (grey) and TDP-43^WT^ (blue) mice at the late symptomatic stage. **(F)** Glycogen concentrations from liver homogenates collected from WT (grey) and TDP-43^WT^ (blue) mice at the late symptomatic stage. All data presented as mean ± SEM; *n=3-4* for all groups. Two-way ANOVA results listed on time course in panels **A** and **C** is the overall significance between genotypes. All bar graphs analysed by a Student’s t-test.
